# Drug use, homelessness and health: responding to the opioid overdose crisis with housing and harm reduction services

**DOI:** 10.1186/s12954-021-00539-8

**Published:** 2021-08-26

**Authors:** Katrina Milaney, Jenna Passi, Lisa Zaretsky, Tong Liu, Claire M. O’Gorman, Leslie Hill, Daniel Dutton

**Affiliations:** 1 Department of Community Health Sciences, Cumming School of Medicine, University of Calgary, TRW 3rd Floor | 3280 Hospital Dr NW, Calgary, AB T2N 4Z6 Canada; 2 School of Public Policy, University of Calgary, Calgary, Canada; 3 Department of Community Health and Epidemiology, Dalhousie University, Halifax, Canada; 4 Alberta Health Services, Edmonton, Canada; 5 HIV Community Link, Calgary, Canada

**Keywords:** Opioids, Homelessness, Harm reduction, Hospital use

## Abstract

**Background:**

Canada is in the midst of an opioid overdose crisis and Alberta has one of the highest opioid use rates across the country. Populations made vulnerable through structural inequities who also use opioids, such as those who are unstably housed, are at an increased risk of experiencing harms associated with opioid use. The main purpose of this study was to explore if there was an association between unstable housing and hospital use for people who use opioids.

**Methods:**

Analysis utilized self-reported data from the Alberta Health and Drug Use Survey which surveyed 813 Albertans in three cities. Hospital use was modeled using a logistic regression with our primary variable of interest being housing unstable status. Chi square tests were conducted between hospital use and variables associated with demographics, characteristics of drug use, health characteristics, and experiences of receiving services to establish model inclusion.

**Results:**

Results revealed a significant association between housing instability and hospital use with unstably housed individuals twice as likely torequire hospital care.

**Conclusions:**

Results highlight the importance of concurrently addressing housing instability alongside the provision of harm reduction services such as safe supply and supervised consumption sites. These findings have significant implications for policy and policymakers during the opioid overdose epidemic, and provide a foundation for future areas of research.

## Background

Canada is currently experiencing a national opioid overdose crisis. Since January 2016, there have been over 15,300 apparent opioid-related deaths across Canada with over 19,300 hospitalizations due to opioid-related poisonings [16]. Between 2013 and 2018, hospitalizations related to opioid poisonings across Canada increased by 27%, while rates of hospitalization and emergency department visits continue to rise [9].

Alberta continues to have some of the highest rates of opioid-related deaths, emergency department visits, and hospitalizations within Canada. In Alberta, 2667 individuals died from an accidental opioid poisoning since January 1, 2016, with over 140 deaths already reported in the first three months of 2020 [14, 15]. Emergency department visits related to opioids and other drug use increased 41% between January 1, 2016 and the third quarter of 2019, while hospitalizations related to opioids and other drug use increased 19% between January 1, 2016 and the third quarter of 2019 [15]. In the last quarter of 2019, Alberta reported over 2470 emergency and urgent care visits associated with opioids and other drug use, with 13% of individuals visiting more than once [15]. Although opioid-related deaths, emergency department visits, and hospitalizations are reported at a broad level, there is a lack of publicly available demographic data. Specifically, there is no government-based reporting on opioid use or overdoses specific to individuals who are unstably housed.

Federal and provincial governments across Canada have taken important steps to support individuals who use opioids across the country by increasing access to treatment, expanding awareness and prevention of opioid-related harms, supporting data collection and research, increasing access to supervised consumption sites, and working to decrease the tainted drug supply including working with international partners and border agents to reduce and seize illegal opioids [16]. However, researchers have argued that more needs to be done including providing access to safer opioids. Although controversial, harm reduction efforts that include access to a safe supply may be the most effective way to reduce overdoses [27].

According to the National Health Care for the Homeless Council (NHCHC), housing is a crucial social determinant of health and a lack of housing, or being unstably housed, is associated with mental health concerns, physical health problems, trauma, greater mortality rates, and substance use disorders [24]. Individuals who are unstably housed are at an increased risk of experiencing opioid use and overdose. For example, Yamamoto et al. [28] found a significantly higher risk of opioid overdose in those who were homeless than those who were housed. Similarly, a study by Doran et al. [10] revealed a significant association between homelessness and opioid overdose. Results from other studies suggest overdose is the leading cause of mortality in the homeless population with rates up to 17 times higher than the general population [4, 5]. In British Columbia, a 2017 report revealed almost 30% of individuals who experienced an overdose reported unstable housing, and those with no fixed address were at a higher risk of experiencing repeated overdoses [7]. Finally, Zivanovic et al. [29] found that unstable housing was independently associated with increased mortality rates, suggesting housing status is an important risk factor to be considered among individuals that use drugs. Thus, there is evidence to suggest there is an association between unstable housing and opioid-related harms.

Individuals who are unstably housed and use opioids often lack access to safe, adequate healthcare and are overrepresented in mental health concerns including substance use, anxiety, and depression [3, 22, 23]. Results from some studies suggest housing instability is associated with higher unmet needs and lower rates of access to a family doctor, resulting in significantly more hospitalizations and visits to emergency departments [18, 19, 21]. Not only does housing instability and a lack of healthcare impact the individual experiencing inequities, the economic impacts are substantial. In 2013, homelessness was estimated to cost the Canadian economy more than $7 billion annually including costs associated with healthcare services [11, 12]. Furthermore, Latimer et al. [22] examined the costs associated with housing homeless individuals with mental health concerns across five Canadian cities and found the average annual cost ranged between approximately $29,000 and $56,000 per person. These authors argue that for every $1 invested in housing and individualized case managed supports, resulted in an average savings of just over $2 in public costs.

To build upon the knowledge surrounding the importance of housing as a critical social determinant of health within the opioid epidemic, the purpose of this study was to examine if housing instability was associated with an increased likelihood of accessing hospital services for problems with emotions, mental health, or alcohol/ drug use with additional variables of interest including demographics, drug use characteristics, health characteristics, and/or experiences receiving services. For the purposes of this study, housing instability and homelessness are used interchangeably.

## Methods

### Participants

We utilized self-reported data from the Alberta Health and Drug Use Survey results (Alberta Health and Drug Use Survey 2017) which surveyed 813 Albertans in three cities, Calgary, Red Deer and Medicine Hat. Participants were recruited through local coalitions of service providers in those cities. Variables are defined in Table 1. Information was collected on: (1) socio-demographics, drug use and health; (2) drug use, risk behaviours, and experience of harm; (3) outcomes related to health status, health service use, and unmet healthcare needs; and (4) acceptability of potential new health services. For the purposes of the current study, the total analytic sample was 432 participants and included those participants who: (1) reported using opioids via injection or noninjection within the six-month period prior to participating in the Alberta Health and Drug Use Survey (opioids included carfentanil, china white, codeine, demerol, fentanyl, heroin, hydrocodone, hydromorphone, methadone, morphine, oxycodone, oxycontin, oxyneo, percocet, speed balls, street methadone, and talwin); and (2) provided a true response (i.e., not ‘Refused’ or ‘Don’t Know’) to questions within the variables of interest for the current study including hospital use, demographics, characteristics of drug use, health characteristics, and experiences receiving services, as defined in Table 1. Ethics approval was obtained through the University of Calgary Conjoint Health Research Ethics Board REB#19-2156.

**Table 1:** Variable definition and coding

Variable of interest	Characteristic/experience absent or infrequent (0)	Characteristic/experience present or frequent (1)
*Outcome variable*		
Hospital use	No—hospital care (overnight or longer) was not used within the last 12 months due to problems with emotions, mental health, or alcohol/drug use	Yes—hospital care (overnight or longer) was used within the last 12 months due to problems with emotions, mental health, or alcohol/drug use
*Demographics*		
Housing unstable	No—did not identify as housing unstable	Yes—identified as housing unstable
Sex	Male participants	Female participants
Ethnicity	Non-indigenous participants	Indigenous participants (first nations, metis, Inuit)
Age (real age)	Participants entered age in years	
Location	Calgary	Medicine hat or red deer
*Characteristics of drug use*		
Had an overdose	No—did not overdose within past 6 months	Yes—did overdose within past 6 months
How often: polydrug use	Used more than 1 type of drug on the same occasion 2–4 times a month or less	Used more than 1 type of drug on the same occasion 2–3 times a week or more
How often: heavily influenced by drugs	Over the past year, are influenced heavily by drugs other than alcohol on a monthly basis or less	Over the past year, are influenced heavily by drugs other than alcohol on a weekly basis or more
How often: irresistible longing to use	Over the past year, you felt you had a longing to use drugs so strong that you could not resist on a monthly basis or less	Over the past year, you felt you had a longing to use drugs so strong that you could not resist on a weekly basis or more
How often: unable to stop use	Over the past year, you have not been able to stop taking drugs once you started on a monthly basis or less	Over the past year, you have not been able to stop taking drugs once you started on a weekly basis or more
How often: neglected tasks due to use	Over the past year, you have neglected to do something you should have due to having used drugs on a monthly basis or less	Over the past year, you have neglected to do something you should have due to having used drugs on a weekly basis or more
How often: need to use in morning	Over the past year, you have needed to use in the morning after heavy drug use the day before on a monthly basis or less	Over the past year, you have needed to use in the morning after heavy drug use the day before on a weekly basis or more
How often: feel guilty due to drug use	Over the past year, you have had guilty feelings or a bad conscience because you used drugs on a monthly basis or less	Over the past year, you have had guilty feelings or a bad conscience because you used drugs on a weekly basis or more
Hurt due to use	No—you or others have not been hurt (mentally or physically) due to your drug use	Yes—you or others have been hurt (mentally or physically) due to your drug use
Others worry due to use	No—relatives, friends, or medical professionals have not been worried about your drug use or said you should stop using	Yes—relatives, friends, or medical professionals have been worried about your drug use or said you should stop using
*Health characteristics*		
Diagnosed with addiction or mental health disorder	No—health professional has not diagnosed you with an addiction or mental health disorder	Yes—health professional has diagnosed you with an addiction or mental health disorder
*Adequacy of non-hospital services*		
Inadequate: could not access	Reported no problem accessing health care services that were perceived as needed. Services include: information about medical treatment, medication or tablets, counselling outside of a hospital, social interventions, skills training, harm reduction services, and medical care for physical health	Reported needing a service but was unable to access the service
Inadequate: did not receive enough	Reported no problem with accessing enough of the health care services listed above, either due to adequate amounts or not requiring them	Reported needing more of a service but was unable to access the amount required

### Procedure

Data were analyzed using Stata. Hospital use was modeled using a logistic regression with our primary variable of interest being unstable housing status. Chi-squared tests were conducted between hospital use and potential confounders. All demographic variables were included in our final model. Variables regarding characteristics of drug use, health characteristics, and experiences receiving services were included if they met a relaxed significance value (*p* ≤ 0.1) and had variance inflation factors (VIF) scores below 2.5 when tested for multicollinearity between independent variables (in a logistic regression on hospital use, not reported). Testing for multicollinearity was conducted due to the multiple variables measuring drug use characteristics. Variables with the highest p-values were removed from the model until the coefficients from the initial model showed a change larger than 20%, which constituted our threshold for confounding. At this point, all variables were left in the model and were considered confounders. Once the final set of variables was established, interaction effects were tested for between demographics and other explanatory variables, only keeping the significant interactions. The adjusted model contained a single significant interaction effect: sex and diagnosis with addiction or a mental health disorder.

## Results

### Sample characteristics by hospital use with Pearson chi-squared results

Table 2 presents sample characteristics by hospital use and results from the chi-squared tests assessing the association between hospital use and demographics, characteristics of drug use, health characteristics, and experiences receiving services.

**Table 2:** Sample characteristics by hospital use with Pearson chi-squared results (*n* = 432)

Characteristic	Total *n* (%)	Hospital use		*P* value
		Yes 183 (42.4%)	No 249 (57.6%)	
*Housing unstable*				
Yes	240 (55.6)	124 (67.8)	116 (46.6)	< 0.001
No	192 (44.4)	59 (32.2)	133 (53.4)	
*Sex*				
Female	150 (34.7)	62 (33.9)	88 (35.3)	0.753
Male	282 (65.3)	121 (66.1)	161 (64.7)	
*Ethnicity*				
Indigenous	141 (32.6)	66 (36.1)	75 (30.1)	0.193
Non-Indigenous	291 (67.4)	117 (63.9)	174 (69.9)	
Age, in years (range)*	37.4 (16–68)	36.3	38.2	0.117
*Location*				
Medicine hat or red deer	224 (51.9)	98 (53.6)	139 (55.8)	0.054
Calgary	208 (48.1)	85 (46.4)	110 (44.2)	
*Drug use characteristics*				
*Had an overdose*				
Yes	137 (31.7)	89 (48.6)	48 (19.3)	< 0.001
No	295 (68.3)	94 (51.4)	201 (80.7)	
*How often: polydrug use*				
2–3×/Week or more	233 (53.9)	114 (62.3)	119 (47.8)	0.003
2–4×/Month or less	199 (46.1)	69 (37.7)	130 (52.2)	
*How often: heavily influenced by drugs*				
Weekly or daily	333 (77.1)	152 (83.1)	181 (72.7)	0.011
Monthly or less	99 (22.9)	31 (16.9)	68 (27.3)	
*How often: irresistible longing to use*				
Weekly or daily	261 (60.4)	121 (66.1)	140 (56.2)	0.038
Monthly or less	171 (39.6)	62 (33.9)	109 (43.8)	
*How often: unable to stop use*				
Weekly or daily	248 (57.4)	113 (61.7)	135 (54.2)	0.118
Monthly or less	184 (42.6)	70 (38.3)	114 (45.8)	
*How often: neglected tasks due to use*				
Weekly or daily	273 (63.2)	140 (76.5)	133 (53.4)	< 0.001
Monthly or less	159 (36.8)	43 (23.5)	116 (46.6)	
*How often: need to use in morning*				
Weekly or daily	291 (67.4)	137 (74.9)	154 (61.8)	0.004
Monthly or less	141 (32.6)	46 (25.1)	95 (38.2)	
*How often: feel guilty due to drug use*				
Weekly or daily	313 (72.5)	143 (78.1)	170 (68.3)	0.023
Monthly or less	119 (27.5)	40 (21.9)	79 (31.7)	
*Hurt due to use*				
Yes	359 (83.1)	160 (87.4)	199 (79.9)	0.040
No	73 (16.9)	23 (12.6)	50 (20.1)	
*Others worry due to use*				
Yes	370 (85.6)	167 (91.3)	203 (81.5)	0.040
No	62 (14.4)	16 (8.7)	46 (18.5)	
*Health characteristics*				
*Diagnosed with addiction or mental health disorder*				
Yes	351 (81.2)	162 (88.5)	189 (75.9)	0.001
No	81 (18.8)	21 (11.5)	60 (24.1)	
*Experience of receiving services*				
*Inadequate access*				
Yes	238 (55.1)	113 (61.7)	125 (50.2)	0.017
No	194 (44.9)	70 (38.3)	124 (49.8)	
*Inadequate amount*				
Yes	246 (56.9)	109 (59.6)	137 (55.0)	0.346
No	186 (43.1)	74 (40.4)	112 (45.0)	

^*^P-value for age is a two-tailed *t*-test

The majority of participants were unstably housed (55.6%), male (65.3%), non-Indigenous (67.4%), and had an average age of 37.4 years (range between 16 and 68). Regarding hospital use, 42.4% of participants reported using hospital care (overnight or longer) within the six months prior to being surveyed. Among those that reported receiving hospital care, 67.8% of participants reported unstable housing compared to those that did not receive hospital care where only 46.6% indicated unstable housing. When looking at location and receiving hospital care, those who received hospital care were more likely to be in Medicine Hat or Red Deer (53.6%).

Of the 432 participants, 31.7% had reported an overdose within the six months prior to taking part in the survey, 53.9% of participants reported using drugs 2–3× per week or more, and 77.1% reported being influenced heavily by drugs weekly or daily, referring to how often a participant used substances other than alcohol. Chi-squared test results revealed multiple significant associations between participants who reported receiving hospital care and characteristics of drug use such as overdosing (*p* ≤ 0.001), frequently neglecting other tasks due to use (*p* ≤ 0.001), frequently needing to use in mornings after heavy usage the night before (*p* = 0.004), frequent polydrug use (*p* = 0.003), frequently being heavily influenced by drugs (*p* = 0.011), others noting they are worried about the participants use (*p* = 0.004), frequently feeling an irresistible longing to use (*p* = 0.038), frequently feeling guilty due to drug use (*p* = 0.023), and believing that themselves or others have been hurt due to their use (*p* = 0.040). In contrast, participants without these higher risk drug use characteristics were proportionally less likely to report receiving hospital care.

Regarding addiction or mental health disorder, 81.2% of participants reported being diagnosed. Chi-squared test results revealed a significant association between diagnosis with addiction or mental health concern and higher likelihood of receiving hospital care (*p* = 0.001).

Of the 432 participants, 55.1% of participants reported they were unable to access a type of service they felt they needed. Chi-squared test results revealed a significant association between receiving hospital care and more unmet needs due to not being able to access a service (*p* = 0.017). 56.9% of participants reported they were unable to access enough services they felt they needed.

### Logistic regression of hospital care

Table 3 reports the results from the logistic regression on unadjusted and adjusted models, including an interaction term accounted for in the adjusted results. In the adjusted model, participants were more likely to use hospitals if they reported unstable housing (Odds ratio (OR): 2.04, 95% confidence interval (CI):1.29–3.21), an overdose (OR: 3.59, 95% CI: 2.21–5.83), or neglecting tasks due to drug use on a frequent basis (weekly or more) (OR: 2.19, 95% CI: 1.28–3.73). After testing for interaction effects between variables, the primary variable of interest for unstable housing did not have any significant interactions with the other variables contained in the model. Therefore, while controlling for all other variables in the model, those who were unstably housed were twice as likely to receive hospital care.

**Table 3:** Logistic regression onto hospital use (*N* = 432)

	Unadjusted			Adjusted		
	OR	95% CI	*P* value	OR	95% CI	*P* value
Housing unstable (1 = yes)	2.409	1.619–3.586	0.000	2.035	1.289–3.214	0.002
Sex (1 = female)	0.937	0.627–1.400	0.753	0.055	0.006–0.474	0.008
Ethnicity (1 = indigenous)	1.319	0.873–1.963	0.193	1.221	0.757–1.968	0.413
Age	0.99	0.97–1.00	0.117	1.00	0.98–1.02	0.69
Location (1 = medicine hat or red deer)	0.686	0.468–1.007	0.054	0.669	0.429–1.046	0.078
Had an overdose (1 = yes)	3.965	2.584–6.083	< 0.001	3.586	2.206–5.829	< 0.001
How often: polydrug use (1 = 2–3 times/week or more)	1.801	1.223–2.663	0.003	1.108	0.675–1.819	0.684
How often: heavily influenced by drugs (1 = weekly or daily)	1.842	1.144–2.966	0.012	1.298	0.722–2.330	0.383
How often: irresistible longing to use (1 = weekly or daily)	1.519	1.023–2.257	0.038	0.753	0.418–1.359	0.347
How often: unable to stop use (1 = weekly or daily)	1.363	0.924–2.011	0.118	0.767	0.439–1.338	0.350
How often: neglected tasks due to use (1 = weekly or daily)	2.839	1.861–4.334	< 0.001	2.187	1.284–3.726	0.004
How often: feel guilty due to drug use (1 = weekly or daily)	1.661	1.069–2.581	0.024	1.094	0.637–1.878	0.745
Others worry due to use (1 = yes)	2.365	1.292–4.329	0.005	1.728	0.850–3.511	0.131
Diagnosed addiction or mental health concern (1 = weekly or daily)	2.449	1.428–4.200	0.001	1.262	0.634–2.513	0.508
Inadequate access (1 = yes)	1.601	1.086–2.361	0.017	1.283	0.815–2.021	0.282
Inadequate amount (1 = yes)	1.204	0.818–1.773	0.346	1.000	0.638–1.569	0.999
Sex * diagnosed	1.331	0.879–2.015	0.177	22.433	2.466–204.064	0.006
How often: need to use in morning (1 = yes)	1.837	1.206–2.797	0.005*	–	–	–
Hurt due to use (1 = yes)	1.747	1.022–2.987	0.041*	–	–	–
Constant				0.001		

^*^Not statistically significant in final adjusted regression model

The interaction effect between sex and having been diagnosed by a professional with an addiction and/or a mental health concern was significant. Among males, the effect of diagnosed status is insignificant and has minimal impact on receiving hospital care; however, among females, those who had a diagnosis of an addiction and/or mental health concern from a professional were 28 times more likely to receive hospital care (OR for females with a diagnosis = exp(interaction) * exp(diagnosis) = (22.443) * (1.261) = 28.26) than their counterparts. These results indicate that while controlling for all other factors within the model, the effect of diagnosis for addiction and/or mental health concerns among males is minimal or negligible in relation to hospital utilization. In contrast to this, diagnosed status among females has a statistically significant and large magnitude association with receiving hospital care.

## Discussion

The main purpose of this study was to examine if housing instability was associated with an increased likelihood to access hospital services for problems with emotions, mental health, or alcohol/drug use specifically amongst individuals who use opioids. Additional variables of interest included demographics, drug use characteristics, health characteristics, and/or experiences receiving services.

Results revealed that being unstably housed was associated with receiving hospital care even after accounting for the additional variables. This finding is similar to previous studies that have found an association between unstable housing status and increased utilization of hospital services [18, 19, 21, 23]. Housing is considered a crucial social determinant of health and being homeless or unstably housed can have negative impacts on health. Homelessness has been associated with high mortality rates, extreme poverty, poor oral and dental health, and chronic conditions such as diabetes, seizures, respiratory problems, Human Immunodeficiency Virus (HIV), and widespread issues with alcohol and drugs [17]. Our findings support the growing evidence base highlighting the importance of housing and recovery-oriented models such as Housing First [12]. Housing First models are rooted in the belief that housing, not compliance or sobriety, is the foundation for improved health and well-being and once housing has been secured, a person can successfully address other areas in their life such as physical health, mental health, substance use, employment, and education [12]. Housing First models adhere to the following five principles: (1) individuals have access to permanent housing with no requirements or conditions, (2) emphasize individual choice and self-determination; (3) focus on recovery within a harm reduction approach; (4) recognize the uniqueness of each individual and their needs once housing is secured; and (5) support individuals to integrate into their community with social supports [12]. Housing First had also been shown to reduce hospitalizations and emergency department visits, thereby decreasing the economic costs associated with homelessness [12]. Furthermore, as Magwood et al. [23] purport, homeless individuals with substance use issues benefit from harm reduction strategies, including Housing First, by improving access to care, reducing opioid overdoses, and preventing or limiting the spread of infectious disease and other chronic conditions. Results from a study of people who use opioids who accessed a Housing First program showed a 93% housing retention rate and 100% of participants accessed overdose prevention education and naloxone while in the program [20].

While access to stable housing could reduce some of the risks associated with overdose, housing in the absence of a variety of harm reduction approaches is not a panacea, particularly for those with complex addictions [26], who are likely at high risk for overdose. Some housing programs utilize a congregate living model with staff and programming onsite while others utilize a ‘scattered site’ model where tenants access a rent supplement and live in a unit in a market housing building. Access to services is limited in these models and previous studies have shown high rates of isolation and loneliness for people in scattered site housing programs [25]. In order to adequately respond to the opioid crisis, harm reduction services must be readily available and include multiple options in addition to community naloxone programs and supervised consumption services. Implementation of safe supply initiatives including regulated and safe opioid distribution are necessary [27]. Access to safe opioids would reduce the likelihood that someone would need to access street drugs that are highly toxic and highly addictive [8].

Although some researchers suggests Housing First models can support the reduction of opioid overdoses in those who are homeless, there are also multiple and persistent barriers to accessing housing programs, including limited capacity to meet the demand and subsequent wait lists. Many people in housing programs are also not receiving the level of heath care they need and are still at high risk for overdose. Supervised consumption sites provide a monitored environment for individuals who use substances and offer referral services such as counselling, social work, and other opioid-dependency treatment options while reducing the transmission of infections and diseases [1]. The Calgary supervised consumption site responded to over 1,800 overdoses between October 30, 2017 and May 31, 2020 and received over 151,000 client visits during this time [2]. A recent study by Jackson showed that each overdose managed at the Calgary supervised consumption clinic saved approximately $1600 per overdose or over $2.3 million in total emergency health costs since the site opened. Bardwell et al. [6] suggest the implementation of supervised consumption sites in emergency shelters, supportive housing buildings, and even mobile and moveable sites may allow for a more flexible and effective response for those who use opioids and experience homelessness. However, while supervision of drug use and overdose reversal reduces mortality, it does not address the underlying issue of street drug toxicity and the harmful effects of drug poisonings. A full spectrum of harm reduction strategies should include low barrier access to safe opioids. Future research should prioritize creation of strategies to reduce systemic barriers to safe supply programs and evaluate the effectiveness of such programs.

Interestingly, our results revealed a significant interaction between sex and having a mental health and/or addiction diagnosis with regard to receiving hospital care. Females who had a mental health and/or addiction diagnosis were 28 times more likely to use the hospital than undiagnosed females, a relationship 22 times higher than the same one in males, suggesting that males and females vary in their likelihood of hospital usage based on if they have received a diagnosis. This indicates that among people who use opioids who receive hospital care, there is an important interplay between females and status of professional diagnoses for addiction and/or mental health. Future research could examine the sex differences associated with opioid use, receiving hospital care, and mental health and/or addiction diagnoses to determine how to best to support varied subpopulations with harm reduction interventions.

## Limitations

This analysis had several limitations that should be taken into consideration. Self-report measures are subject to bias and open to interpretation by participants. Since the Alberta Health and Drug Use Survey focused on collecting survey data from individuals actively accessing services and agencies, individuals that were not actively accessing services were not captured within this analysis resulting in a potentially non-representative sample. Similarly, previous research suggests homelessness has been associated with a lower likelihood of seeking treatment, which could also indicate a nuanced subset not covered within this analysis [13].

The analytic sample may not be generalizable because participants were excluded from the analytic sample if they: (1) did not identify as either male or female due to low representation of non-binary participants among the collected surveys; (2) only used non-opioid drugs, in order to focus specifically on opioid drug use; and/or (3) could not provide clear answers to the questions, or did not know or refused to answer questions. Finally, while many variables and potential explanatory factors were controlled for or assessed as confounders it is possible that there are additional unobserved explanatory factors not contained within this analysis. Finally, the cross-sectional approach in our study limits what can known about causality. Longitudinal research that includes access to administrative health data and/or a control groups could clarify differences in hospital usage for a variety of sub groups.

## Conclusion

Opioid overdoses in Canada continue to be a significant public health crisis and individuals who are unstably housed are extremely susceptible to overdose. Unstably housed individuals who use opioids are more likely to utilize hospital services, which impacts both their individual health and wellness while having significant economic and social costs on society. These findings highlight the importance of considering Housing First programs in conjunction with a full spectrum of harm reduction services including supervised consumption services and access to safe opioids. This is particularly important within the context of increasing drug toxicity, drug poisonings and overdoes.

## Data Availability

The datasets generated and/or analyzed during the current study are not publicly available due to share ownership with not for profit community-based organizations but may be available from the corresponding author on reasonable request.

## Abbreviations

CI: Confidence interval
HIV: Human immunodeficiency virus
NHCHC: National health care for the homeless council
OR: Odds ratio
VIF: Variance inflation factors
